# Abnormal functional connectivity of the nucleus accumbens subregions mediates the association between anhedonia and major depressive disorder

**DOI:** 10.1186/s12888-023-04693-0

**Published:** 2023-04-21

**Authors:** Yanqin Hu, Chaoqi Zhao, Houfeng Zhao, Juan Qiao

**Affiliations:** 1 Department of Psychiatry, First Clinical College, Xuzhou Medical University, Xuzhou, 221000 China; 2 Department of Psychiatry, the Affiliated Xuzhou Oriental Hospital of Xuzhou Medical University, Xuzhou, 221000 China; 3 Department of Medical Psychology, Second Clinical College, Xuzhou Medical University, Xuzhou, 221000 China

**Keywords:** Anhedonia, Functional connectivity, Major depressive disorder, Nucleus accumbens subregions

## Abstract

**Background:**

The nucleus accumbens (Nac) is a crucial brain region in the pathophysiology of major depressive disorder (MDD) patients with anhedonia. However, the relationship between the functional imaging characteristics of Nac subregions and anhedonia remains unclear. Thus, this study aimed to investigate the role of resting-state functional connectivity (rsFC) of the Nac subregions between MDD and anhedonia.

**Methods:**

We performed resting-state functional magnetic resonance imaging (fMRI) to measure the rsFC of Nac subregions in 55 MDD patients and 30 healthy controls (HCs). A two-sample t test was performed to determine the brain regions with varying rsFC among Nac subregions between groups. Then, correlation analyses were carried out to investigate the relationships between the aberrant rsFC of Nac subregions and the severity of anhedonia. Furthermore, we constructed a mediation model to explain the role of the aberrant rsFC of Nac subregions between MDD and the severity of anhedonia.

**Results:**

Compared with the HC group, decreased rsFC of Nac subregions with regions of the prefrontal cortex, insula, lingual gyrus, and visual association cortex was observed in MDD patients. In the MDD group, the rsFC of the right Nac shell-like subregions with the middle frontal gyrus (MFG)/superior frontal gyrus (SFG) was correlated with consummatory anhedonia, and the rsFC of the Nac core-like subdivisions with the inferior frontal gyrus (IFG)/insula and lingual gyrus/visual association cortex was correlated with anticipatory anhedonia. More importantly, the functional alterations in the Nac subregions mediated the association between anhedonia and depression.

**Conclusions:**

The present findings suggest that the functional alteration of the Nac subregions mediates the association between MDD and anhedonia, which provides evidence for the hypothesis that MDD patients have neurobiological underpinnings of reward systems that differ from those of HCs.

## Background

Major depressive disorder (MDD) has become one of the leading causes of disease burden worldwide, and its lifetime prevalence is approximately 3.4% [1, 2]. As one of the recognized core symptoms of MDD, anhedonia includes a wide range of reward processing defects, such as reward learning, evaluation, expectation, motivation, effort expenditure, and consummatory pleasure [3–6]. For MDD patients, anhedonia is a strong predictor of psychosocial functioning improvement, mediating between the severity of depressive symptoms and improvement in social functioning [7]. In addition, MDD patients with anhedonia have a more severe course of illness and a higher risk of suicide [8].

The deficit of reward circuits is a core mechanism of anhedonia, which originates from the dopaminergic mesolimbic pathway in the ventral tegmental area (VTA) and to the Nac, amygdala, and hippocampus of the ventral striatum (VS) [9–11]. Neuroimaging has found that the striatum plays an important role in both anticipatory pleasure (“wanting”) and consummatory pleasure (“liking”) [12]. And the concepts of “wanting” and “liking” correspond to the hypothesis that different subregions of the striatum have corresponding functions [13, 14]. When naltrexone is used to inhibit the function of opioid receptors, dorsolateral prefrontal cortex (dlPFC)-striatum connections are enhanced [15, 16], and top-down inhibition of the prefrontal cortex (PFC) to the striatum is achieved [17]. Similarly, after the reward “liking” stage of MDD patients, low activation was observed in the striatum [18] as was excessive activation of dlPFC [19]. This evidence is related to abnormal signalling of opioids that mediate “liking”, which is consistent with the neurobiological hypothesis that the striatum plays a key role in hedonic processes [20, 21] and supports optogenetic studies in animals showing that overactivation of PFC can inhibit the striatal response to reward and lead to anhedonia [19].

The Nac, located in the ventral part of VS, is an important brain region that regulates motivational learning [22, 23] and mainly mediates the hedonic perception of rewards, which is related to reward evaluation and expectation [24]. Neuroimaging studies have found aberrant alterations in the reward system in MDD patients. Structural magnetic resonance imaging studies have found that the volume of left Nac in MDD patients is larger than that in HCs [25]. Resting-state fMRI studies have indicated that rsFC between the Nac and extensive cortical regions, such as the orbitofrontal cortex (OFC) and anterior cingulate cortex (ACC), are reduced in MDD patients [26]. Previous findings support the hypothesis that the reward systems work as a whole and that key structures cannot work in isolation from the whole [27]. Abnormal reward processing is thought to disrupt the VTA-Nac pathway in MDD patients [28]. That is, MDD may change reward circuits that are associated with the VTA-Nac pathway so that patients fail to feel pleasure and motivation [29, 30].

Recent studies have explored the functional character of the Nac on a subregional level, revealing that Nac subregions have different functions in an indivisional’s pleasure experience process. The Nac can be divided into the core-like part and shell-like part. Studies have found that the core-like subdivision receives projections from the mediolateral OFC and prelimbic medial prefrontal cortex (mPFC) [31, 32], and the shell-like subdivision receives projections from the ventral medial prefrontal cortex (vmPFC) [33]. The two subregions have different inputs and outputs, indicating that they contribute differentially to goal-directed behaviours [34, 35]. Animal studies have found that the Nac core-like subdivision is related to appetite controls and responses to aversive motivation [36], goal-directed behaviour, instrumental learning, and motivation [37, 38], while the shell-like subdivision is associated with the integration of motivational valence and novelty [39, 40].

Neurobiological studies have found that reward processing procedures include desire, anticipation, effort to attain reward, consummatory pleasure, and cognitive aspects of learning [5]. Whereas anticipatory pleasure is more closely related to reward motivation and goal-directed behaviours (i.e., “wanting”), consummatory pleasure may more accurately reflect pleasure in the present moment and satisfaction after every obtained reward (i.e., “liking”) [41]. This distinction is critical for identifying the specificity and the mechanisms of reward-related deficits in MDD and schizophrenia [42]. Anticipatory pleasure and consummatory pleasure represent different types of pleasure with different brain regions and neural circuits [43]. We divided the Nac into two functionally distinct subregions, the core and the shell, by the 2-cluster solution [44]. Anticipatory anhedonia may be mediated by the Nac core-like subdivision, while consummatory anhedonia is mediated by the Nac shell-like subdivision [45–47]. Previous empirical work on anhedonia in depression has mainly focused on consummatory anhedonia, while there have been relatively few studies on anticipatory anhedonia. Questionnaire and laboratory measures of anhedonia have also emphasized the consummatory phase [48]. Therefore, consummatory anhedonia was once considered to be the main cause of anhedonia in MDD. A recent study of MDD patients supports that anticipatory pleasure is as flawed as consummatory pleasure [49]. More importantly, anticipatory anhedonia and consummatory anhedonia show a dissociated pathophysiological basis [50]. To comprehensively understand the relationship between the consummatory and anticipatory dimensions of anhedonia and the rsFC of Nac subregions in MDD patients, we administered the Temporal Experience of Pleasure Scale (TEPS) to all participants to measure the severity of their anhedonia as this assessment can be used to distinguish between anticipatory anhedonia and consummatory anhedonia [51]. The innovative point of this study was the division of Nac into subregions and the investigation of the correlation between the two dimensions of anhedonia and the rsFC of Nac subregions. Then, we hope to evaluate clinical subjects with a two-dimension scale to provide evidence for finding potential biological markers of MDD with specific anhedonia.

In this study, we first hypothesized that the rsFC of Nac subregions would be different between the MDD and HC groups, Second, we hypothesized that there were correlations between the rsFC of the Nac core-like subdivision and anticipatory anhedonia as well as the rsFC of the Nac shell-like subdivision and consummatory anhedonia in MDD patients, which differed from that in HCs. And this variance may be associated with a different neurobiological basis in Nac subregions within the two groups [52], indicating that MDD may damage the normal function of Nac subregions in the reward circuit. Third, we hypothesized that the altered rsFC of Nac subregions play mediating roles between group and the severity of anhedonia. As the largest variable factor, MDD may indirectly regulate the severity of anhedonia by changing the rsFC of the Nac subregions.

## Materials and methods

### Participants

A total of 55 patients with depression and 30 healthy controls were included in this research. All of the MDD patients were inpatients in the Affiliated Xuzhou Oriental Hospital of Xuzhou Medical University who met the following criteria: [1] the diagnostic criteria of the major depressive disorder according to the Diagnostic and Statistical Manual of Mental Disorders, fifth edition (DSM-V) criteria; [2] right-handed; [3] age in the range of 18 to 55 years old; and [4] generally normal intelligence. The MDD patients who met the following criteria were excluded: [1] previous or existing mental disorders other than MDD; [2] secondary depressive episodes caused by organic mental disorders or other diseases; [3] neurodegenerative diseases, such as brain trauma, cerebrovascular diseases, and other organic cerebral diseases; [4] history of severe cardiac dysfunction, renal insufficiency, hepatic diseases, poorly controlled diabetes or other major somatic diseases; [5] pregnant, breastfeeding and preparing for pregnancy; and [6] contraindications for a magnetic resonance imaging (MRI) scan. The HCs recruited were matched with the MDD patients in age, sex, and education years and lived in the same or similar place as the MDD patients. HCs all met exclusion criteria the same as MDD patients. This study was approved by the Medical Ethics Committee of Xuzhou Medical University Affiliated East Hospital. All subjects signed informed consent forms.

### Behavioural assessment

All subjects completed clinical and behavioural assessments. The Montgomery-Asberg Depression Rating Scale (MADRS) was used to assess the severity of depression. The lower the score, the lower the depression severity. TEPS was used to assess the severity of anhedonia. The lower the score, the higher the anhedonia severity. Chan [53] designed the TEPS for Chinese people in the context of Chinese culture based on the TEPS, which was verified [54, 55]. The scales above were administered on the day of the fMRI examination by 2 psychiatrists with professional training.

Differences in demographic and other data between the HC group and the MDD group were compared using the two-sample t test and chi-square test.

### Image acquisition

We conducted fMRI with Siemens 3.0T on all subjects at Xuzhou Medical University Affiliated East Hospital. During scanning, subjects were required to remain awake, keep their eyes closed, keep their heads fixed, and lay quietly on the examination bed without thinking actively. Structural T1-weighted images were acquired by the gradient recalled echo sequence. The parameters were set as follows: repetition time (TR)/echo time (TE) = 1900 ms/2.58 ms, field of vision (FOV) = 250 × 250 mm^2^, matrix = 256 × 256, layer number = 176, layer thickness = 1 mm, and voxel size 3 × 3 × 3 mm, and scanning time = 4 min 18 s. If no abnormalities were found, resting-state functional scans were performed. Resting-state fMRI images were obtained by conducting a gradient-recall echo-planar imaging (GRE-EPI) pulse sequence with the following parameters: TR/TE = 3000 ms/40 ms, FOV = 240 mm×240 mm, 32 layers, matrix size = 64 × 64, layer thickness = 4 mm, time point = 135, and scanning time = 6 min 56 s.

### Functional imaging data preprocessing and preliminary data analysis

Neuroimaging data were transformed by MRIcron (http://www.mccauslandcenter.sc.edu/mricro/mricron). Then, we obtained the remaining 130 time point data after discarding the first five time images. Functional MRI data were preprocessed using the CONN toolbox (Cognitive and affective neuroscience laboratory, Massachusetts Institute of Technology, Cambridge, MA, USA; www.nitrc.org/projects/conn) and SPM12(Wellcome Department of Imaging Neuroscience, London, UK; www.fil.ion.ucl.ac.uk/spm) [56], running on MATLAB R2013b (MathWorks). The spatial preprocessing steps included slice-timing correction, realignment, and two-step registration by using indirect segmentation and normalization. Then, the standard brain was spatially smoothed by 4 mm ×4 mm ×4 mm, and head motion < 2 mm or < 2° was required for inclusion. In order to remove unwanted motion, physiological, and other artifactual effects from the blood oxygen level-dependent (BOLD) signal, linear regression was performed with three different sources of possible confounders, including white matter and cerebrospinal fluid masks (5 dimensions each), scrubbing and realignment parameters (12 regressors: 6 motion parameters + 6 first-order temporal derivatives), and the effect of rest (2 regressors: 1 motion parameters + 1 first-order temporal derivatives) [56, 57]. In addition, we used a seed-based approach to obtain the rsFC values after adjusting the filter to 0.01–0.08 Hz to avoid possible confounding effects.

**Fig. 1:**
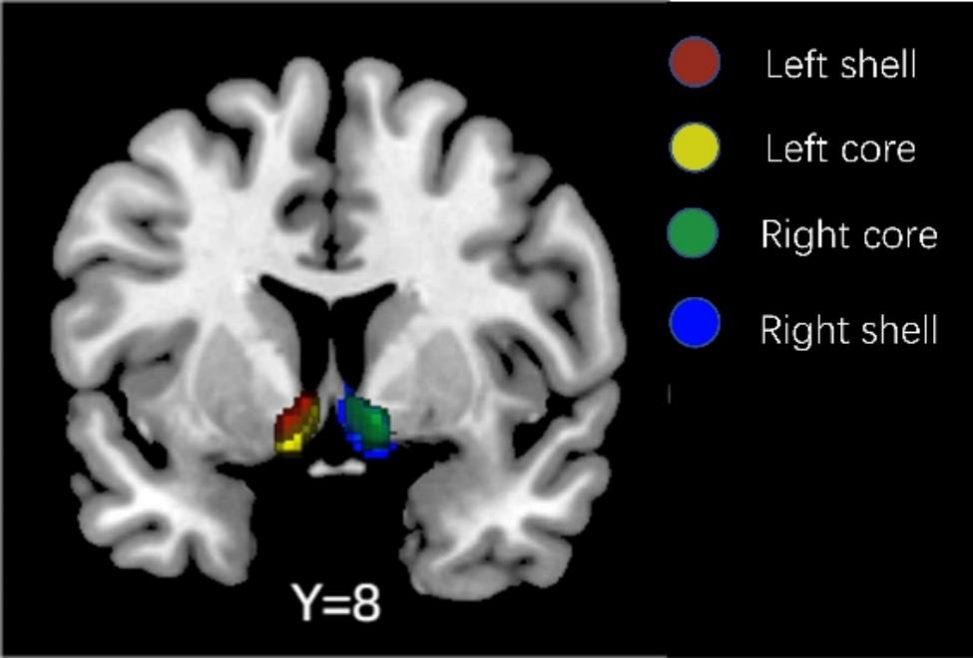
The four Nac subregions characterized by Cartmell et al. [44]

Compared to a previous study [58], Cartmell et al. leveraged the larger number of subjects in their dataset, minimizing uncertainty generated during the acquisition and processing pipeline to produce a greater signal [44]. The core-like and shell-like subdivisions of Nac were defined based on a probabilistic atlas of Nac subregions [44]. Then, the bilateral core-like and shell-like subdivisions of Nac were chosen as seeds that corresponded to 4 subregions (the left core-like subdivision, the right core-like subdivision, the left shell-like subdivision, and the right shell-like subdivision) (Fig. 1).

The first-level analysis included bivariate correlations between seeds and all voxels throughout the whole brain. Then, we obtained brain maps for each individual. In order to verify the accuracy of the NAc subregions segmentation, we conducted one-sample t tests on the rsFC maps of the two groups, respectively (voxel-wise p < 0.001; cluster-level family-wise error (FWE) p < 0.05). In addition, paired-samples t-tests were conducted to compare the differences in rsFCs between the ipsilateral NAc subregions in HCs (voxel-wise p < 0.001; cluster-level FWE p < 0.05). A two-sample t test in the CONN toolbox was used to compare the rsFC values between the two groups and to identify the brain regions with differences between MDD patients and HCs (thresholded at a whole-brain P < 0.001 uncorrected voxel threshold and cluster-level FWE p < 0.05). BrainNet Viewer (https://www.nitrc.org/projects/bnv/) was used to report significantly different brain regions.

### Correlation analysis

To investigate the correlation between the rsFC values and TEPS scores, we conducted Spearman and Pearson correlation analyses (Benjamini/Hochberg method was used to control the false discovery rate, adjusted p < 0.05). Then, the rsFC values and group were used as the independent variables, and the TEPS score was the dependent variable, while sex, age, and education years were controlled. Multiple linear regression with the “Enter” method was conducted using SPSS 26 software to explore the influencing factors of anhedonia.

### Mediation analysis

We performed bootstrap analyses that were generated from 5,000 bootstrapped samples controlling for age, sex, and years of education to test the mediating role of the rsFC of Nac subregions in the relationships between group and the TEPS scores on Model 4 of PROCESS V4.0 by Andrew F Hayes’ in IBM SPSS Statistics version 26.0. First, group was defined as the independent variable (X), while the TEPS score was defined as the outcome variable (Y). Then, we tested whether the indirect effect of X on Y (a × b) was statistically significant. If the 95% CI did not cross 0, the mediation effect existed. In addition, we tested whether the direct effect of X on Y (c) is statistically significant. The relationship between X and Y was completely mediated if the 95% CI crossed 0, or the relationship between X and Y was mediated partially.

## Results

### Demographic and clinical information

As shown in Table 1, we did not find significant differences in age, sex, or education years (p > 0.05) between the groups of participants. The MADRS score was higher and the TEPS, anticipatory pleasure dimension of TEPS (TEPS_C), and anticipatory pleasure dimension of TEPS (TEPS_A) scores were lower in the MDD group than in the HC group (p < 0.001). In conclusion, MDD patients had significantly more severe anticipatory and consummatory anhedonia than HCs.

**Table 1:** Demographic and clinical characteristics

Characteristics	MDD(N = 55)	HC(N = 30)	*Z*/*T*/*χ* Value	*P* Value
Sex(male/female) Age (years) Education (years) MADRS TEPS TEPS_A TEPS_C	18/37 35.18 ± 12.41 11.47 ± 3.23 33.04 ± 5.67 62.98 ± 10.26 25.78 ± 5.68 34.07 ± 5.64	9/21 36.83 ± 9.80 11.90 ± 1.936 1.97 ± 1.03 86.73 ± 4.62 36.87 ± 3.06 47.43 ± 3.66	0.067 -0.629 -0.661 39.456 -14.662 -11.691 -13.191	0.796^a^ 0.531^b^ 0.510^b^ 0.000^b^ 0.000^b^ 0.000^b^ 0.000^b^

Abbreviations: *MDD* major depression disorder, *HC* healthy control, *MADRS* Montgomery-Asberg Depression Rating Scale, *TEPS* the Temporal Experience of Pleasure Scale, *TEPS_A* anticipatory pleasure dimension of the Temporal Experience of Pleasure Scale, *TEPS_C* consummatory pleasure dimension of the Temporal Experience of Pleasure Scale

^*a*^*p* value obtained by chi-squared test

^*b*^*p* value obtained by two-sample t test

### RsFC analyses of each Nac subregion

**Fig. 2:**
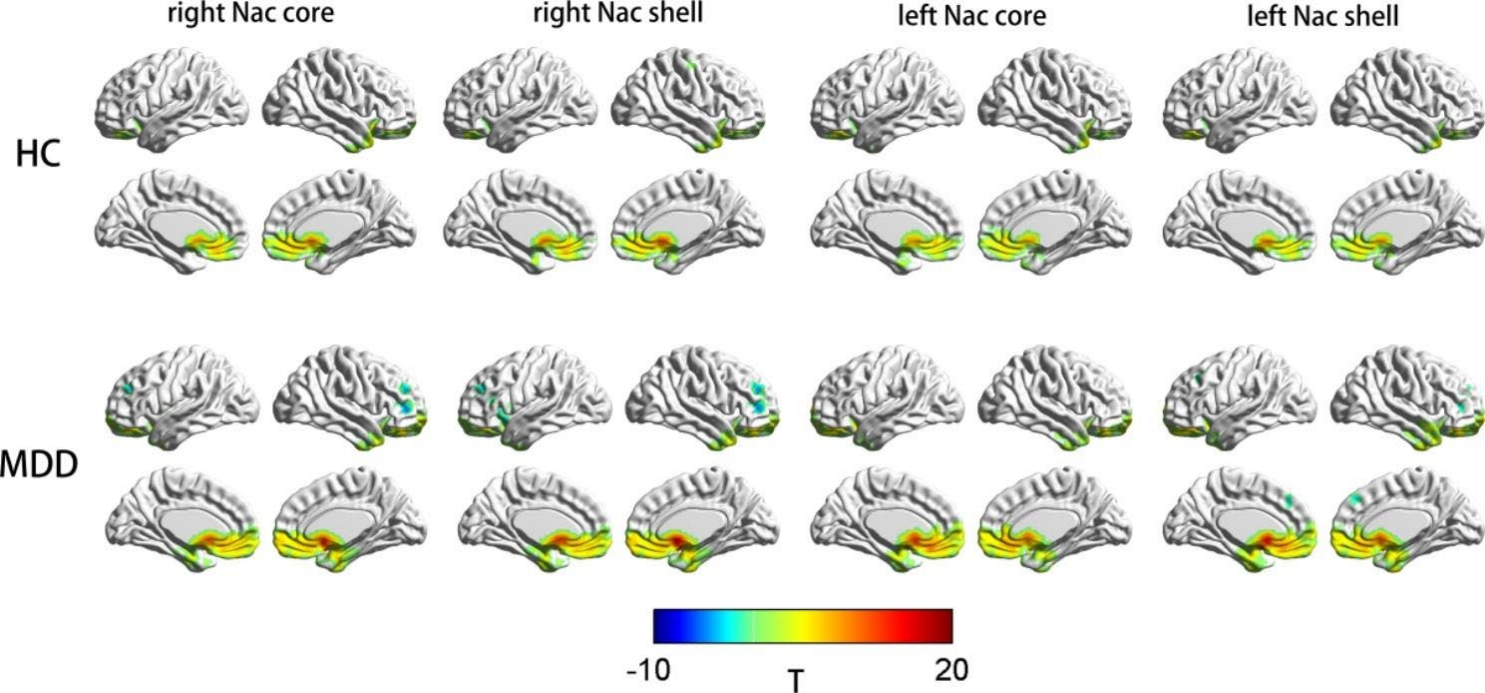
Spatial distributions of the rsFC of the four Nac subregions between the HC group and the MDD group. rsFC data were projected onto the images of the brain using BrainNet Viewer (https://www.nitrc.org/projects/bnv/). The colour bar scale represents t values

One-sample t test analyses revealed that positive rsFCs with the four Nac subregions and the regions of the frontal lobe, temporal lobe, ACC, subcallosal gyrus, basal ganglia, OFC, and parahippocampal gyrus in both the MDD group and HC group (Fig. 2).

**Fig. 3:**
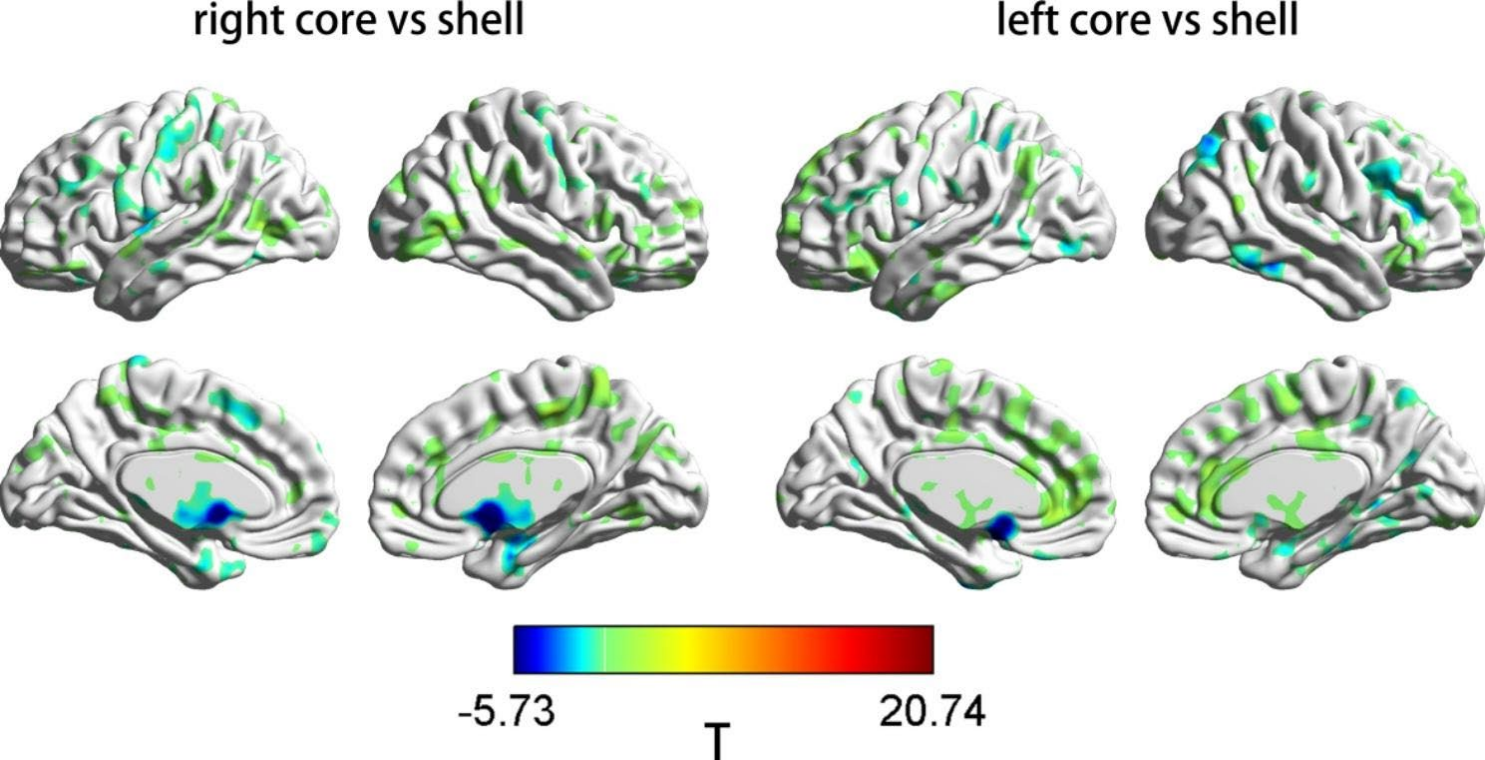
Regions showing significant differences in rsFC with the Nac core-like

In the HC group, paired-samples t test analyses revealed that the rsFC of Nac core-like subdivisions with the regions of the frontal lobe and ACC was stronger than that of shell-like subdivisions, and the rsFC of Nac core-like subdivisions with the regions of subcallosal gyrus and parahippocampal gyrus was weaker than that of shell-like subdivisions. These findings are consistent with those of previous studies [52, 58, 59] (Fig. 3).

subdivision compared with the ipsilateral Nac shell-like subdivision in the HC group. Warm colours represent the regions showing stronger rsFC with the Nac core-like subdivision than with the ipsilateral shell-like subdivision. Cool colours represent the regions showing weaker rsFC with the Nac core-like subdivision than with the ipsilateral shell-like subdivision. Images were created using BrainNet Viewer (https://www.nitrc.org/projects/bnv/). The colour bar scale represents t values.

**Fig. 4:**
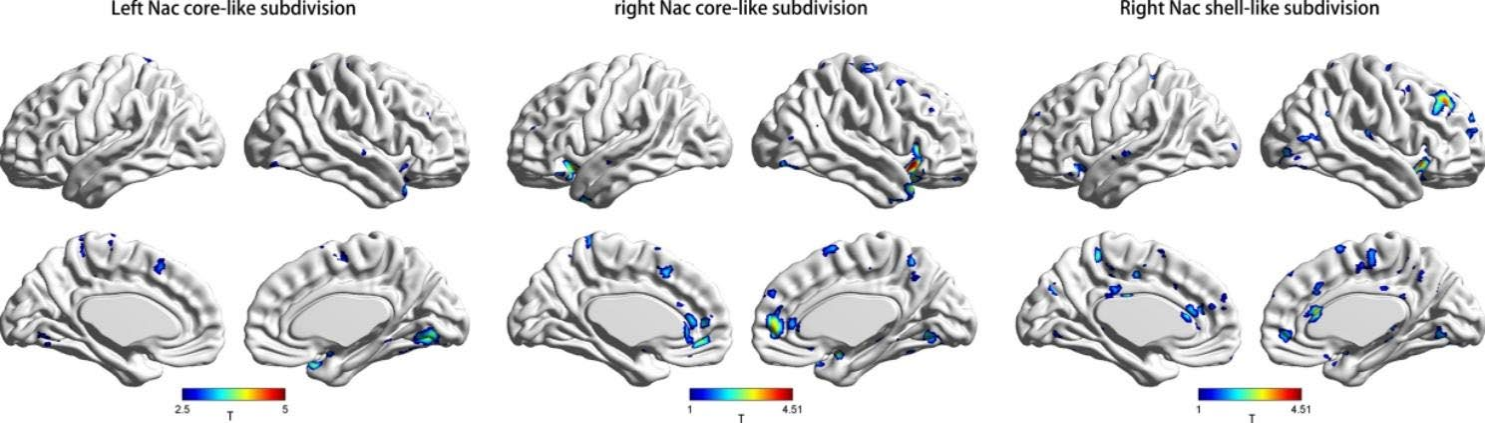
Regions showing significant differences in rsFC with the Nac subregions between the MDD group and HC group. Warm colours represent the regions showing stronger rsFC of Nac subregions in the MDD group than in the HC group. Cool colours represent the regions showing weaker rsFC of Nac subregions in the MDD group than in the HC group. The images were created using BrainNet Viewer (https://www.nitrc.org/projects/bnv/). The colour bar scale represents t values

Compared with the HC group, we found that decreased rsFC between the left Nac core-like subdivision and right lingual gyrus/visual association cortex in the MDD group (FWE p < 0.001); decreased rsFC between the right Nac core-like subdivision and right IFG/insula in the MDD group (FWE p = 0.003); and decreased rsFC between the right Nac shell-like subdivision and right MFG/SFG in the MDD group (FWE p = 0.009) (Fig. 4; Table 2).

**Table 2:** Group differences in rsFC between the MDD group and the HC group

Seed region	Cluster location	Hemisphere	Peak (MNI)			Number of voxels	t-value	p-value
			X	Y	Z			
Core_L	Lingual gyrus/Visual association-cortex	Right	27	-66	-3	95	4.4014	< 0.001
Core_R	IFG/insula	Right	30	15	-9	65	5.3071	0.003
Shell_R	MFG/ SFG	Right	18	36	36	54	4.4789	0.009

Abbreviations: *MDD* major depressive disorder, *HC* healthy control, *MNI* Montreal Neurological Institute, *Core_R* right Nac core-like subdivision, C*ore_L* left Nac core-like subdivision, *Shell_R* right Nac shell-like subdivision, *IFG* inferior frontal gyrus, *SFG* superior frontal gyrus, *MFG* middle frontal gyrus

### Correlation between anhedonia severity and imaging characteristics

**Fig. 5:**
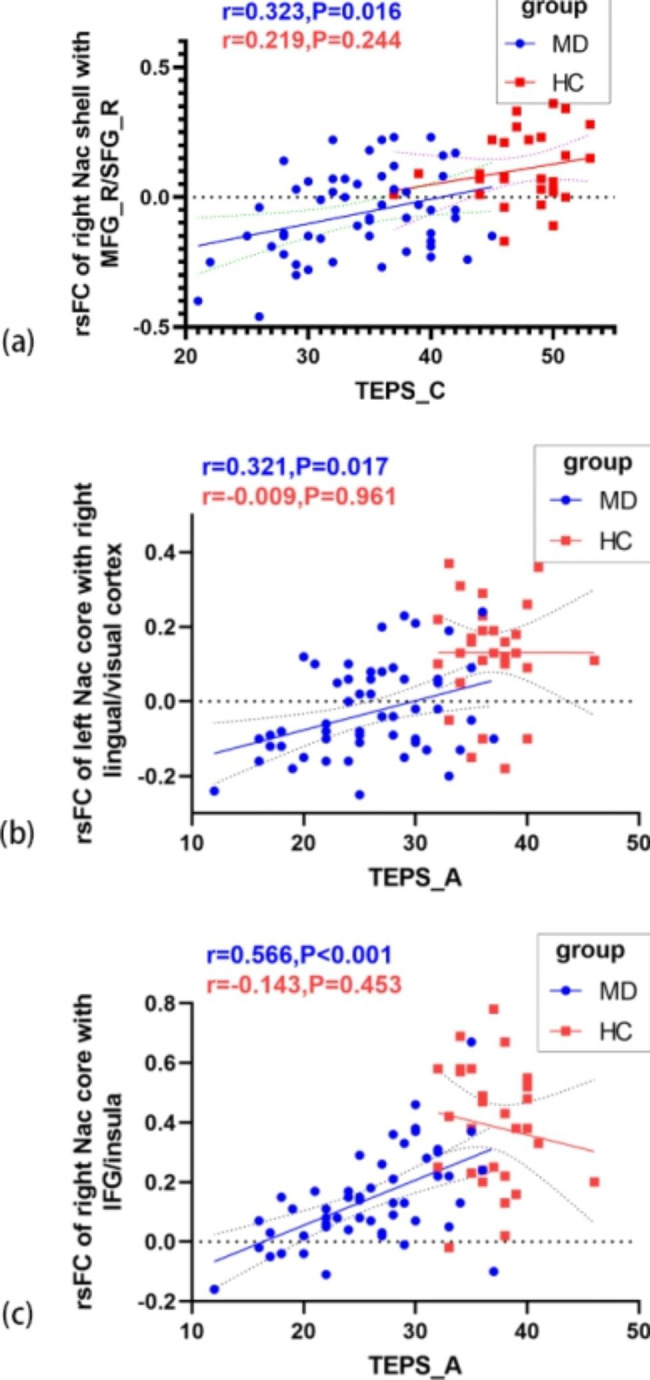
Scatter plots of the correlation between significantly different rsFC and the anhedonia scale in both groups. (a) Positive correlation between rsFC of the right Nac shell-like subdivision and TEPS_C score. (b) Positive correlation between rsFC of the left Nac core-like subdivision and TEPS_A score. (c) Positive correlation between rsFC of the right Nac core-like subdivision and TEPS_A score. (MD, major depressive disorder; HC, healthy control; TEPS_C, consummatory pleasure dimension of the Temporal Experience of Pleasure Scale; TEPS_A, anticipatory pleasure dimension of the Temporal Experience of Pleasure Scale; IFG, inferior frontal gyrus; SFG_R, right superior frontal gyrus; MFG_R, right middle frontal gyrus)

This study revealed that TEPS_C scores were positively correlated with rsFC values between the right Nac shell-like subdivision and right MFG/SFG (r = 0.323, p = 0.016, adjusted p = 0.048), and TEPS_A scores were positively correlated with rsFC values between the right Nac core-like subdivision and right IFG/insula (r = 0.566, p < 0.001, adjusted p < 0.001) and with rsFC values between the left Nac core-like subdivision and right lingual gyrus/visual association cortex (r = 0.321, p = 0.017, adjusted p = 0.034) in the MDD group (Fig. 5). However, we did not find any significant correlation in the HC group. All of the above are shown in Fig. 4.

### Multiple linear regression analysis of the factors of anhedonia

Multiple linear regression analysis revealed that group and rsFC values were statistically significant (Tables 3 and 4). It is worth noting that rsFC values were less significant when group was taken as an independent variable in multiple linear regression models (Tables 3 and 4). When we analyzed the MDD and HC groups separately, the rsFC of the Nac subdivisions became significant (p < 0.001). The results showed that abnormal rsFC values of Nac core-like subdivisions and the right Nac shell-like subdivision were independent factors of anticipatory anhedonia and consummatory anhedonia, respectively (Tables 3 and 4). Variance inflation factor (VIF) scores were all less than 2, which indicated that these variables had acceptable multicollinearity.

**Table 3:** Results of multiple linear regression analysis for anticipatory anhedonia

	B	Std.error(SE)	Beta	t	95% CI	p	VIF
**Sex**	1.196	1.074	0.077	1.113	(-0.942, 3.334)	0.269	1.036
**Age**	-0.044	0.046	-0.07	-0.955	(-0.136, 0.048)	0.343	1.165
**Education**	0.106	0.192	0.042	0.553	(-0.277, 0.489)	0.582	1.219
**Group**	-7.553	1.392	-0.502	-5.427	(-10.323, -4.782)	0	1.832
**Core_L**	8.697	4.073	0.179	2.135	(0.588, 16.806)	0.036	1.505
**Core_R**	8.88	2.995	0.253	2.965	(2.918, 14.842)	0.004	1.558

R^2^ adjusted = 0.608

Abbreviations: *Core_R* rsFC values of the right Nac core-like subdivision with the IFG/insula, *Core_L* rsFC values of the left Nac core-like subdivision with the right lingual gyrus/visual cortex, *VIF* variance inflation factor

**Table 4:** Results of multiple linear regression analysis for consummatory anhedonia

	B	Std.error(SE)	Beta	t	95% CI	p	VIF
**Sex**	2.727	1.097	0.157	2.487	(0.544, 4.91)	0.015	1.013
**Age**	0.072	0.048	0.102	1.504	(-0.023, 0.168)	0.137	1.178
**Education**	0.293	0.192	0.102	1.525	(-0.089, 0.676)	0.131	1.142
**Group**	-11.48	1.22	-0.678	-9.411	(-13.908, -9.052)	0	1.321
**Shell_R**	10.018	3.376	0.214	2.967	(3.297, 16.738)	0.004	1.323

R^2^ adjusted = 0.670

Abbreviations: *Shell_R* rsFC of the right Nac shell-like subdivision with the right MFG/SFG, *VIF* variance inflation factor

### Testing for the mediator

**Fig. 6:**
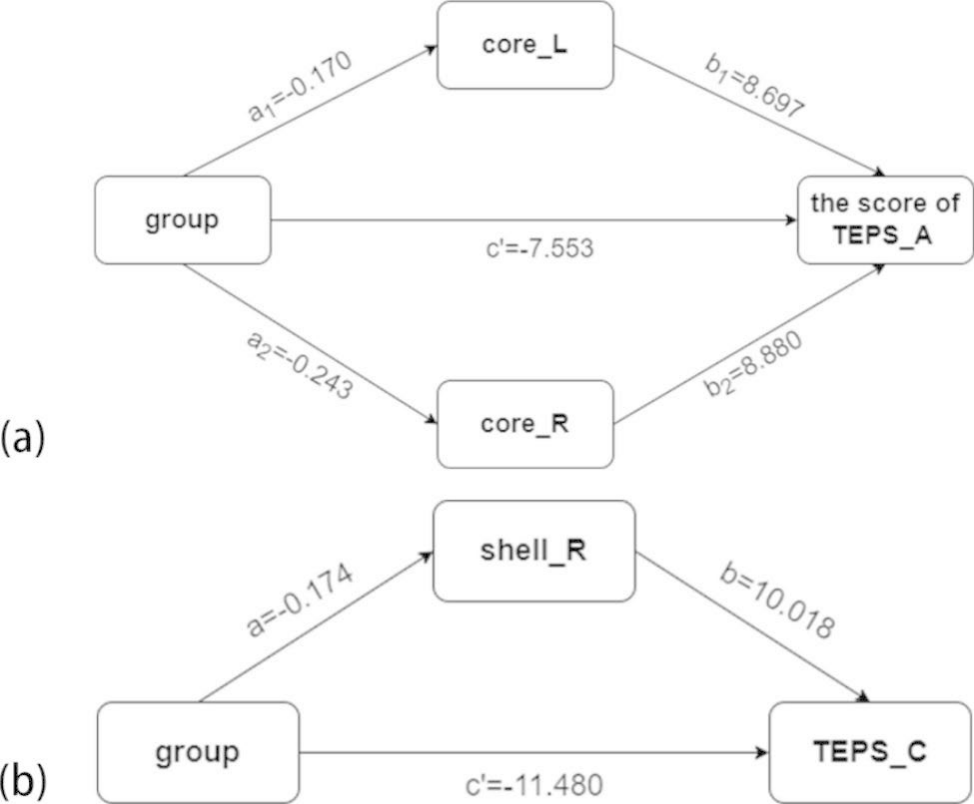
Mediation models for the effect of rsFC on the relationship between group and anhedonia. (a) Mediation model with regression path coefficients of the rsFC of bilateral core-like subdivisions as mediators of the relationship between group and anticipatory anhedonia. (b) Mediation model with regression path coefficients of the rsFC of the right shell-like subdivision as a mediator of the relationship between group and consummatory anhedonia. (core_L, rsFC value of the left Nac core-like subdivision with the right lingual gyrus/visual cortex; core_R, rsFC value of the right Nac core-like subdivision with the IFG/insula; TEPS_A, anticipatory pleasure dimension of the Temporal Experience of Pleasure Scale; shell_R, rsFC value of the right Nac shell-like subdivision with the right MFG/SFG; TEPS_C, consummatory pleasure dimension of the Temporal Experience of Pleasure Scale)

The results showed (Table 5) that the 95% CI of the TEPS_C indirect effect (-0.185, -0.025) and direct effect (-13.847, -9.072) as well as the 95% CI of the TEPS_A indirect and direct effect did not cross 0. This finding indicated that the rsFC values of Nac subregions play partial mediating roles between MDD and anhedonia. According to the results above, the two mediation models we built presented path coefficients associated with the models (Fig. 6). Finally, the mediating effect accounted for 13.213% (a1×b1/c), 19.258% (a2×b2/c) and 13.155% (a×b/c) of total effect in Models (a) and (b), respectively.

**Table 5:** Mediation model and the mediation effect of rsFC

	Effect	SE	t	p	95% CI
**TEPS_C** Indirect effect of X on Y (a x b) Direct effect of X on Y (c’) Total effect of X on Y	-1.739 -11.480 -13.219	0.717 1.220 1.121	- -9.411 -11.795	- 0 0	(-3.255, -0.442) (-13.908, -9.052) (-15.449, -10.989)
**TEPS_A** Indirect effect of X on Y (a1 x b1) Indirect effect of X on Y (a2 x b2) Direct effect of X on Y (c’) Total effect of X on Y	-1.478 -2.154 -7.553 -11.185	0.759 0.937 1.392 1.120	- - -5.427 -9.987	- - 0 0	(-3.147, -0.183) (-4.230, -0.517) (-10.323, -4.782) (-13.414, -8.956)

We also noticed that the bilateral rsFC of Nac core-like subdivisions both affected the mediation model(a). Then, we performed Spearman correlation analysis for the two sides of the subdivision. However, no significant correlation between bilateral Nac core-like subdivisions was found (p > 0.05). We also performed a mediation analysis separately, which showed that the rsFC of the left Nac core-like subdivision and the rsFC of the right Nac core-like subdivision still had partial mediation effects (effect accounted for 15.832% and 21.239%, respectively). The right Nac core-like subdivision had higher weight than the left as the mediator.

Additionally, the strength of the rsFC of Nac subregions played partial mediating roles in our models (Fig. 6). MDD partially influenced the severity of anhedonia by changing the rsFC of Nac subregions, revealing that MDD has a regulatory effect on the neural basis of anhedonia [52]. Then, our study revealed that MDD may affect anticipatory pleasure through the core-like subdivision related neural basis while affecting consummatory pleasure through the shell-like subdivision. This evidence was consistent with the incentive salience theory [45–47].

## Discussion

In this study, fMRI was used to investigate the differences in the rsFC of Nac subregions between MDD patients and HCs in Xuzhou city and surrounding areas in China. Compared with HCs, rsFC decreased in the bilateral core-like subregion and right shell-like subdivisions of Nac in the MDD group. And the abnormal rsFC values of Nac subregions were negatively correlated with anhedonia in the MDD group. However, we found no correlation in the HC group, indicating that MDD patients and HCs have different neurobiological bases in the “liking” and “wanting” processes of pleasure. The correlation between both the rsFC of the Nac core-like subdivision and shell-like subdivision and the severity of anhedonia in MDD patients is regulated by depression itself. In addition, mediating models demonstrate that decreased rsFC of Nac subdivisions is a significant mediator between MDD and anhedonia, suggesting that MDD can indirectly affect anhedonia by altering the functional connections of Nac subregions.

We found that the rsFC of the left Nac core-like subdivision with the right lingual gyrus/visual association cortex was reduced in MDD patients, which was negatively correlated with anticipatory anhedonia. Anatomically, the lingual gyrus extends to the fusiform gyrus and joins the parahippocampal gyrus to form emotion-limbic circuits, which are associated with visual memory recall and emotional processing [60]. The lingual gyrus is associated with high-level visual processing and visual memory [61]. There is significantly reduced grey matter volume in the prefrontal lobe, limbic system, striatum, lingual gyrus, and fusiform gyrus in MDD patients [62]. During emotional facial processing, grey matter structural [62–64] and functional abnormalities [65] are found in the lingual gyrus and fusiform cortices within MDD patients. Impaired lingual gyrus and fusiform gyrus under the control of the prefrontal network are thought to underlie the onset of MDD and may manifest as deficits in visual memory, working memory, and emotional bias [62, 66]. Compared with non-melancholic MDD patients, a recent study reveals that the decreased long-range positive rsFC in the right lingual gyrus in melancholic MDD patients [67]. Decreased rsFC of the lingual gyrus and fusiform gyrus in MDD patients is also observed [68]. This evidence suggests that the lingual gyrus and its related brain areas are closely associated with anhedonia in MDD patients. Clinically, deep brain stimulation of the nucleus accumbens (DBS-Nac) acts as a suppressor of neuronal activity [69, 70], which can improve depressive anhedonia [71, 72]. In addition, DBS-Nac can be feasible in the treatment of severe alcohol use disorder, which disrupts the normal rsFC between the Nac and the visual association cortex [73]. That is, DBS-Nac may disturb the “wanting” (anticipatory motivation) and “learning” loops of alcohol addiction. We speculate that DBS-Nac might change the rsFC between the left Nac core-like subdivision and major regions of emotion-limbic circuits as well as the visual association cortex, which may be related to anticipatory anhedonia. Moreover, greater sensory reactivity in the visual cortex could predict depressive relapse [74], and anhedonia is a predictor factor for episodes of depression as well. These results provide potential evidence that there is a correlation between the Nac core-like subdivision and the visual cortex. Based on the role of the lingual gyrus and visual association cortex in hedonic and reward processing and their functional and structural abnormalities, we hypothesized that decreased rsFC between the Nac left core-like subdivision and right lingual gyrus/visual association cortex may indicate that the reward loop is destroyed in MDD patients. This might lead to motivation and subsequent learning dysfunction in these individuals.

Moreover, we found that the rsFC of the right Nac core-like subdivision with right IFG/insula [75] was reduced in MDD patients, which was negatively correlated with anticipatory anhedonia. Similar alterations in the IFG have been observed in sleep deprivation experiments [76]. The IFG is thought to regulate the function of Nac [77]. The IFG is closely associated with the inhibition of hedonic-cue response [78]. At the network level, the IFG belongs to the executive control network [79], which also regulates the reward network system. Furthermore, the decreased rsFC between the right IFG and right Nac core-like subdivision means decreased functional synchronization between the executive control and reward networks and leads to an abnormal ability to process and control negative reward information. The functional characteristics of IFG are related to eudaimonic well-being, whereas the OFC is related to positive affect [80]. In addition, a recent study found that MDD patients shared decreased dynamic regional phase synchrony values in the OFC extending to the insula compared to HCs [81], and the right insula extended to the right IFG. Pleasant music might significantly activate the interaction of Nac with the insula and OFC [82], indicating that the OFC is correlated with the insula and IFG in hedonic and reward states. Studies have demonstrated that the insula can also affect the function of Nac, suggesting that the anticipation process of gain/loss involves an ‘alerting’ signal (thalamus) that converges with interoceptive information (insula) to shape action selection programs in the VS [83]. In addition, the insula is an important brain area in the Nac-DBS treatment of psychiatric disorders [84] and is involved in regulating autonomic and physiological responses to rewarding and emotional stimuli [85]. Insula and limbic structures may reveal some correlation during a clinical trial of repeated ketamine treatment for MDD patients [86]. Given that MDD patients may not be able to exclude psychomotor activity or thinking during MRI examination, the brain regions identified are not completely consistent with the differential brain regions indicated by previous studies [52]. However, in terms of the function of brain regions, we can explain the association among the insula, IFG, and medial OFC in reward processing-related processes. The medial OFC is one critical region of the emotion regulation neural systems and rewarding systems [45, 87, 88]. Both the Nac and OFC have been previously thought to represent the expected value of a cue to guide reward-learning behaviour [18, 89]. During the reward anticipation stage, the OFC [90, 91] in MDD patients shows low activation. The over-activation of OFC will project to the Nac core-like subdivision when MDD patients enjoy pleasure. Hence, the decreased rsFC of Nac-OFC provides support for MDD patients with consummatory anhedonia. All evidence supports that the IFG, OFC, and insula are involved in hedonic and reward processing. Based on the role of IFG and insula in hedonic and reward processing and our findings, we speculate that the decreased rsFC between the right Nac core-like subdivision and IFG/insula in MDD patients indicates that the reward system in MDD is disrupted when patients experience anticipatory anhedonia.

Although altered rsFC of bilateral Nac core-like subdivisions are not exactly the same in the MDD group, both of them were negatively correlated with anticipatory anhedonia in accordance with our findings. In our perspective, the rsFC of Nac core-like subdivisions may be relatively separate from related brain areas and unified on the defect of anticipatory pleasure [92, 93]. The correlation of the left core-like subdivision is lower than that of the right, which means that rsFC on the right may have higher weight than that on the left in reflecting the severity of anticipatory anhedonia in MDD patients.

Moreover, we found that rsFC decreased between the right Nac shell-like subdivision and right MFG [75, 94]/SFG [94] in MDD patients, which was negatively correlated with consummatory anhedonia. The SFG and MFG are involved in affective and self-referential processes, which are closely related to satisfaction with life [95]. The MFG is one part of the dlPFC and is closely correlated with eudemonic well-being [96]. Many studies have revealed that structural or functional abnormalities in the SFG and MFG are common in MDD patients [97–101]. In addition, MDD patients with anhedonia exhibited decreased regional homogeneity in SFG [102]. Cerebral functional nodal characteristics of the left SFG were associated with the severity of consummatory anhedonia in MDD patients with severe anhedonia [103]. And abnormal voxel-mirrored homotopic connectivity values in the SFG in MDD patients with anhedonia were found in a recent study [104]. During the reward outcome stage, the right MFG showed decreased activation in MDD patients compared to controls [105]. According to our findings and previous studies [102–105], we speculate that there are abnormal functional changes in both right MFG and SFG can reflect the severity of consummatory anhedonia. Furthermore, the rsFC between Nac shell-like subdivision and right MFG/SFG may be a potential biomarker to predict the severity of consummatory anhedonia.

Almost 70% of MDD patients experience significant anhedonia. In the reward circuit, the Nac acts as a hub that integrates information. Animal studies have found that knockout and inactivation of tachykinin precursor-1 neurons in the NAC lateral shell-like subdivision can induce consummatory anhedonia-like behaviour, indicating that the neurotransmitters delivered by the Nac might affect the function of downstream brain regions in MDD patients [72]. A positron emission tomography study revealed that abnormally high D2/3 receptor availability in the VS in MDD patients is correlated negatively with the severity of motivational anhedonia [106]. There was a particularly close relationship among MDD, aberrant function of Nac, and anhedonia. To our knowledge, few previous studies have explored whether the altered rsFC of the Nac subdivisions might be mediators of the relationship between group and the severity of anhedonia and, if so, to what extent the relationship is mediated. In our study, the altered abnormal rsFC of the Nac subdivisions was a partial mediator of the relationship between group and the severity of anhedonia. One major mechanism that links the group and the severity of anhedonia may be altered rsFC of Nac subdivisions. In this topic, we provided a new perspective and a potential explanation, that is, MDD might damage the motivation or the capacity for hedonic activity and nurturing one’s well-being by indirectly affecting the rsFC of Nac-specific subregions in addition to a direct effect. However, we did not find a potential mediating association among consummatory anhedonia, group, and rsFC of the left shell-like subdivision, suggesting that the pathophysiological basis of bilateral shell-like subdivisions in MDD patients might be isolated.

In the end, this study had some limitations. First, the sample size is not large enough to avoid negative results. In this regard, future studies with larger sample sizes are needed. Second, some MDD patients had taken antidepressant drugs before hospitalization. Although we collected data within three days after admission, we could not rule out the influence of psychiatric drugs on the brain function of these patients. Third, we evaluated the participants’ pleasure experience by a self-report scale, and the results may have been affected by participants’ emotional state at the time of assessment and memory bias. Future research is encouraged to use more comprehensive reward-related scales and tasks to comprehensively assess the subtypes of anhedonia across more dimensions. Last, this study only collected fMRI data of MDD patients within 3 days after admission (baseline level); thus, data after treatment are lacking. It is necessary to determine the score reduction rate after discharge and even long-term longitudinal follow-up studies. In summary, future studies need to be more carefully designed, with larger sample sizes to facilitate our understanding of the neural mechanisms.

## Conclusion

Taken together, we found that abnormal rsFC of Nac subregions in MDD patients compared to that in HCs. There are different neurobiological bases of reward circuits between the MDD and HC groups [52]. Moreover, abnormal rsFC of the Nac subregions is significantly associated with the severity of anhedonia, which is a mediator between group and the severity of anhedonia. Our study might extend the knowledge about functional alterations of specific Nac subregions in the pathophysiology of MDD and the neurobiological underpinnings of MDD patients with anhedonia, which can clearly differentiate them from healthy people. The functional alterations in Nac subregions have the potential to be imaging biomarkers of anhedonia in MDD patients, which can help make clinical diagnosis and treatment more accurate. In the future, longitudinal studies are needed to clarify these inferences.

## Data Availability

The data in this study is not publicly available due to ethical approval and confidentiality agreements made with participants, but are available from the corresponding author upon reasonable request.

## List of Abbreviations

ACC: Anterior cingulate cortex
BOLD: Blood oxygen level-dependent
DBS-Nac: Deep brain stimulation of the nucleus accumbens
dlPFC: Dorsolateral prefrontal cortex
DSM-V: Diagnostic and Statistical Manual of Mental Disorders, fifth edition
fMRI: Functional magnetic resonance imaging
FOV: Field of vision
FEW: Family-wise error
GRE-EPI: Gradient-recall echo-planar imaging
HCs: Healthy controls
IFG: Inferior frontal gyrus
MADRS: Montgomery-Asberg Depression Rating Scale
MDD: Major depressive disorder
MFG: Middle frontal gyrus
MNI: Montreal Neurological Institute
mPFC: Medial prefrontal cortex
Nac: Nucleus accumbens
OFC: Orbitofrontal cortex
PFC: Prefrontal cortex
rsFC: Resting-state functional connectivity
SFG: Superior frontal gyrus
TE: Echo time
TEPS: Temporal Experience of Pleasure Scale
TEPS_A: Anticipatory pleasure dimension of the Temporal Experience of Pleasure Scale
TEPS_C: Consummatory pleasure dimension of the Temporal Experience of Pleasure Scale
TR: Repetition time
VIF: Variance inflation factor
vmPFC: Ventral medial prefrontal cortex
VS: Ventral striatum
VTA: Ventral tegmental area
